# Implementation of a Chief Resident Selection Process Designed to Mitigate Bias: Lessons Learned

**DOI:** 10.7759/cureus.48116

**Published:** 2023-11-01

**Authors:** Rachel J Katz-Sidlow, Kirsten L Roberts, Dacone A Elliott, Edward E Conway, Jr.

**Affiliations:** 1 Pediatrics, Jacobi Medical Center, Albert Einstein College of Medicine, Bronx, USA

**Keywords:** chief resident selection, graduate medical education, inclusion, diversity, chief residents

## Abstract

Background: Chief residency selection processes are often opaque and beset by bias, which can result in disparities in who is selected for this important role. As a chief residency can lead to future academic and/or leadership positions, efforts to increase diversity in academic medicine and physician leadership may be aided by an inclusive chief resident (CR) selection process designed to mitigate bias.

Objective: To implement and evaluate the acceptability of a CR selection process that is inclusive and designed to mitigate bias.

Methods: In the 2021-2022 academic year, we designed and implemented a CR selection process aligned with published strategies known to mitigate bias in academic recruitment. The four-step opt-out CR selection process included a nomination survey, structured interviews, a clinical review, and a holistic review of each candidate. Each step was clearly delineated, assigned a specific number of points, and scored on a designated rubric. The candidates with the highest and second-highest number of points were awarded the two CR positions. Our selection process excluded examination scores and precluded consideration of “fit” between the selected CRs, as these are known sources of potential bias. In January 2023, we surveyed our department to obtain post-implementation feedback and to assess satisfaction with the process, before repeating the process for 2022-2023.

Results: Survey response rates were 47% (14/30) for residents and 29% (18/63) for departmental faculty. The majority of responding residents (64%) and faculty (100%) were satisfied with the CR selection process, finding it fair and inclusive. Nearly 80% of residents and 100% of faculty wished to repeat the process in 2022-2023.

Conclusions: An inclusive CR selection process utilizing strategies to mitigate bias was feasible, and acceptable to residents and faculty. We recommend that residency training programs make efforts to implement CR selection practices that are inclusive and aim to mitigate bias.

## Introduction

Chief residency is a considered an honor, and may facilitate a future career in academia and/or professional leadership positions for those selected [1-6]. At the same time, the selection process for the chief resident (CR) role varies across programs, is often opaque to the stakeholders and can be vulnerable to various types of biases, which may include affinity or in-group bias, gender bias, and racial and ethnicity biases, among others [1-3,7]. Efforts to increase diversity in academic medicine and physician leadership may be aided by an inclusive process that facilitates the selection of a diverse pool of CRs [1-3].

Those most influential in CR selection include program directors and associate program directors, current chiefs, coordinators, and the departmental chair [1,8]. CRs are often chosen for their “fit” with each other and with departmental leadership, which may lead to in-group bias, with influencers unconsciously favoring candidates most similar to themselves [1]. Some programs consider examination scores in the CR selection process; however, this introduces another known source of bias, as standardized test scores may be influenced by family income level and access to educational resources, and therefore disadvantage those from lower socioeconomic strata. [1,9]. In addition, test scores do not necessarily translate to excellence in the clinical competencies desired of a CR [2,10].

To mitigate bias in CR selection, other approaches are needed. Published recommendations for reducing bias in CR selection include creating a diverse selection committee, seeking input from diverse stakeholders, an open call for applications, and the exclusion of standardized exam scores [1,2,7]. Further strategies recommended to reduce bias in academic recruitment and hiring include the use of structured interviews, holistic review (HR), and explicitly defined selection criteria/rubrics [1,2,11,12]. In addition, it is recommended that those involved in the selection process receive anti-bias training [2,11,12].

To our knowledge, our paper is the first to describe the implementation and evaluation of a CR selection process designed to mitigate bias.

## Materials and methods

Jacobi Medical Center is a public hospital in Bronx, NY, affiliated with the Albert Einstein College of Medicine. Our pediatrics department’s two future CRs (postgraduate year (PGY)-4 year) are selected annually from the 15-member PGY-2 class. 

Starting in December 2021, we worked under a tight timeline to design and implement a new process for CR selection, as the new CRs would be announced in March 2022. We began with a literature review, from which we developed three foundational documents: a list of strategies known to mitigate bias in recruitment/hiring; a formal job description for the chief residency role, which was informed by input from departmental leadership and current CRs, and was distributed to all residents and faculty; and a list of desired qualities for the CR role (DQCR). This list was intended to mitigate in-group bias by focusing faculty and residents on the desired qualities for the position, rather than on their personal affinity for any of the candidates. Based on the literature and input from departmental leadership, our final DQCR list consisted of six qualities: leadership, organizational skills, communicator, excellent clinical skills, advocate for our diverse group of residents, approachable [1,13].

We then developed a four-step selection process, with each step assigned a specific number of points. An assessment rubric was designed for each step, indicating how the points would be determined (Table 1).

**Table 1:** CR selection process design ACGME: Accreditation Council for Graduate Medical Education; HR: Holistic review; VS: Vision statement; CR: Chief resident

Selection process step	Eligible points	Rubric description
Resident and faculty nomination survey	Up to 15 points	Candidates were ordered based on the total number of nominations received. The candidate with the highest number of nominations received the full 15 points, the candidate with the second highest number of nominations received 14 points, etc. Tied candidates each received the same number of points (the higher number).
Structured interviews	Up to 20 points	Up to 5 points awarded for each of 4 areas: Motivation, Leadership Skills, Interpersonal Skills, Communication Skills. The scoring rubric defined what was needed to achieve the full score of 5 points for each of these 4 areas.
Clinical excellence	Up to 9 points	ACGME’s 9 point-scale and anchors were used to assess 3 clinical skills: Organize and Prioritize Patient Care, Clinical Reasoning, and Patient Management. The scores for each of these 3 areas were averaged for each rubric, and across all major clinical divisions, to obtain a final score of up to 9 points.
HR	Up to 20 points	Up to 5 points awarded for each of 4 areas reviewed: VS, CV, a quantitative review of comments received on the nomination survey, and a qualitative review of those comments. The scoring rubric defined what was needed to achieve the full score of 5 points in each of these areas.

The residents who achieved the highest and second-highest number of total points in this process would be named our department’s CRs.

The process was approved by the departmental Education Committee and disseminated to all faculty and residents through meetings and emails. Feedback was elicited from faculty and residents to refine the final process.

Candidates were required to write a ≤ 500 word “Vision statement” (VS) describing their interest in and vision for the CR role. Residents were told that the VS would be viewed only by the Chief Resident Selection Committee (CRSC) during the HR.

The CRSC was comprised of a faculty representative from our department’s major clinical divisions along with representation from departmental leadership. All major divisions were included so that candidates' clinical strengths could be fairly represented. In addition, the seven-member CRSC self-identified across diverse racial, ethnic, and gender groups, with members from three generations and all available institutional academic ranks. 

To facilitate an inclusive selection process, we began with an open call for self-nominations. However, as we approached the deadline, only two residents had self-nominated. To remove potential barriers to self-nomination, we quickly pivoted to an opt-out approach. Residents were informed that they would all be listed on the nomination survey, and were actively encouraged to continue through the selection process. Residents could opt-out by contacting program leadership or by not submitting a VS by the deadline.

Faculty and resident nomination survey

An on-line survey instrument was designed with all PGY-2 residents listed. For each resident, respondents could select “I nominate this resident” or “Insufficient interaction.” There were no required responses. An optional text box was included for respondents to add resident-specific comments for the CRSC to consider. In order to mitigate in-group or affinity bias, respondents were asked to nominate all candidates they believed embodied the six DQCR, and to have these qualities in mind during the survey. Before distribution, this survey was pilot tested for ease of use, question clarity and time to completion. The survey was circulated to all departmental faculty and residents. One reminder email was sent during the week the survey was open.

Respondents were required to enter an email address to ensure the survey was completed only once by each respondent. Responses were later de-identified and grouped. Comments were imported into a Microsoft Word document and sorted by resident.

Candidates were ordered based on the total number of nominations each received. The candidate who received the highest number of nominations received the full 15 points, the candidate with the second highest number of nominations received 14 points, etc. Tied candidates received the same number of points (the higher of the 2 possible numbers). 

Structured interviews

All residents who submitted a VS by the deadline moved forward to the structured interview. Interviews were conducted by a three-person CRSC subcommittee, who met as a group with each candidate individually. All interviewers were trained on the concept of a structured interview and on the scoring rubric. All had received prior anti-bias training, and additionally all interviewers reviewed the Association of American Medical Colleges's (AAMC's) online seminar on unconscious bias in the recruitment process [14]. A list of standardized interview questions was developed by the group. Each question was assigned to one of the three interviewers, who asked the same questions, in the same order, at each interview. Questions included asking the candidate to reflect on how they would encourage an inclusive residency environment. Following the 30-minute interview, each interviewer scored the candidate’s responses on a rubric, with a maximum of 20 points available from this step. 

To minimize conformity bias, scoring was done individually and immediately after the interview, before any discussion of the candidate. Individual interviewer scores were averaged to determine the final score for each candidate.

Clinical review

As recommended, our process excluded standardized test scores [1,2]. At the same time, faculty feedback recommended a clinical assessment of candidates; others have similarly found that faculty value strong clinical skills in CR selection [13]. We could not find guidance in the literature on how to incorporate candidates’ clinical performance into CR selection. We prepared a rubric to measure clinical excellence; the rubric consisted of the Accreditation Council for Graduate Medical Education's (ACGME's) nine-point scale and anchors for the following three clinical skills, which were determined to be most relevant for our CR role: Organize and Prioritize Patient Care, Clinical Reasoning, and Patient Management [15].

The CRSC representative from each of the five major clinical divisions completed the form with group input from division members. Each rubric was averaged and then combined with the averages from all five divisions to determine a final score. 

Holistic review

The HR was conducted by the CRSC. All CRSC members had received varied prior training in bias mitigation and engaged in a discussion on this topic before conducting the HR. During the HR, each candidate’s VS and CV were evaluated for experiences and attributes that were deemed relevant for the CR role, and which aligned with our department’s DQCR. These findings were then scored on a rubric. In addition, the CRSC completed a quantitative and qualitative analysis of the written comments submitted on the faculty/resident survey for each candidate. To receive maximum points on the qualitative review, comments needed to be uniformly positive and aligned with the DQCR. A one-point deduction was given for each comment coded as “negative”. To minimize conformity bias, CRSC members individually scored each of these sections before any group discussion occurred. Individual scores were averaged to determine a final HR score.

Our process worked as intended. Pivoting to an opt-out strategy doubled the pool of candidates moving forward in the process. The rubrics for each step worked well to elicit a sub-score after each of the four steps. These results were then easily tallied to obtain a final score, which resulted in the selection of the two CRs for our department for the year 2023-2024.

In January 2023, we surveyed our department to determine satisfaction with the 2021-2022 selection process and for feedback prior to re-implementation. A Google Forms survey was sent to all 2022-2023 PGY-2 residents and departmental faculty. The survey also asked respondents to rate their agreement levels regarding whether the CR selection process was fair, transparent and inclusive. A separate survey was sent to the 2022-2023 PGY-3 class, who had undergone the CR selection process. PGY-3 residents were additionally asked for the reasons they may have chosen to opt out of the selection, and what was the most important, or "deciding factor" that led them to opt-out. Before distribution, the survey was pilot tested for ease of use, question clarity and time to completion. Survey responses were anonymous. The survey was open for one week, and one reminder email was sent during that time period.

The project was approved by the Institutional Review Board of the Albert Einstein College of Medicine as an exempt study.

## Results

The survey response rate was 47% (14/30) for residents and 29% (18/63) for faculty. The majority of responding residents (64%) and faculty (100%) were satisfied with the 2021-2022 CR selection process, finding it fair and inclusive. Nearly 80% of residents and 100% of faculty wished to repeat the process in 2022-2023. 

Although the majority of residents and faculty found the process transparent, this category received the lowest scores. Suggestions from survey respondents included clearer reminders and descriptions of the selection process, and publicizing the salary and benefits for the CR role. Some residents requested the ability to read the candidate’s VSs.

Reasons for eligible residents opting out are listed in Table 2.

**Table 2:** CR selection process post-implementation survey results *Noted as deciding factor by at least one respondent CR: Chief resident; VS: Vision statement

Percentage of respondents by cohort who strongly agree/agree with the following statements:		
	Residents	Faculty
Process was fair	64%	100%
Process was inclusive	71%	100%
Process was transparent	57%	95%
I was satisfied	64%	100%
Class of 2023 reasons for opting out of the selection process (multiple responses allowed): 47% response rate (7/15)		
	Percent of respondents who strongly agree/agree	
Other plans after graduation	57%	
Didn’t want to run against a colleague who wanted the CR role*	57%	
Believed I wasn’t as qualified as my peers*	29%	
Wasn’t interested in the role*	29%	
CR selection process too daunting	14%	
VS required too much effort	0%	
Family responsibilities	0%	

## Discussion

Our CR selection process worked as intended, was feasible, and was well-accepted by faculty and residents. Until the final points were tallied, it was not possible to know which two residents would be awarded the CR positions. The sense that the “the process” would select the candidates, rather than a small group of individuals, provided a sense of equanimity. Also, since our CRs were selected solely by the number of total points received after all four steps, consideration of a candidate’s “fit” with their co-chief was eliminated as a potential source of bias. Our CRs are expected to be able to work well together, regardless of which two individuals are selected.

In order to mitigate in-group bias in our selection process, we initially utilized an open call for applications; however, there was a reluctance among our residents to self-nominate. Prior research has similarly found hesitancy among residents to self-nominate for the CR position [7]. This reluctance among our residents may have related to underlying imposter syndrome, which can manifest during residency, as 29% of our survey respondents stated they believed they were not as qualified for the role as their peers [16]. Importantly, underrepresented in medicine physicians may be affected by imposter syndrome and/or insufficient mentoring, which may present barriers to self-nomination [17,18]. After recognizing the paucity of self-nominations, we pivoted to an opt-out process as described. This change in strategy doubled our pool of candidates. To maximize the number of diverse candidates, we recommend considering an opt-out approach as an alternative to an open call for self-nominations.

Survey results indicated that some residents opted out because they did not want to compete with a peer who wanted the position. During the 2021-2022 season, it became known through open discussion among the residents which peers were active in the selection process. For 2022-2023, residents were encouraged to keep their candidacy confidential. We believed that a confidential process would be more inclusive by encouraging the candidacy of residents who may have otherwise opted out in order not to compete with a peer.

Other enhancements for 2022-2023 included publicizing the CR salary and benefits, as well as a resident town hall and emails to faculty and residents to reiterate the selection process. In addition, residents who had definite plans after graduation were allowed to opt-out earlier, and were not included on the nomination survey. Finally, the CRSC anti-bias training was standardized, with all members required to review the AAMC video [14].

Limitations include that our study occurred in a single pediatrics department with a 15-resident class size, in an institution serving an underserved and vulnerable population and committed to diversity. Our findings may, therefore, not be directly applicable to other types of programs. In addition, our survey response rates were 47% for residents and 29% for faculty. It is possible that non-responding individuals may have felt differently than those who responded. We had opted to limit survey reminders in the context of competing pressures on house staff and attending time, along with a concern for departmental survey fatigue. Nevertheless, a 25-30% response rate can be typical for emailed surveys [19].

## Conclusions

In recent years, there have been calls for residency programs to reconsider how they select CRs, and to create more inclusive processes that mitigate bias. We designed and implemented a new process for selecting our department’s CRs that was aligned with published strategies known to mitigate bias in academic recruitment. We found our new CR selection process to be both feasible and well-accepted by residents and faculty. We recommend that training programs make efforts to design CR selection processes that are inclusive and aim to mitigate bias. As the chief residency may lead to future academic careers and professional leadership roles, facilitating the selection of a diverse cohort of CRs is consonant with larger efforts to enhance diversity in academic medicine and physician leadership.
